# Abstracts From JADPRO Live at APSHO 2016

**Published:** 2017-01-01

**GAYLORD NATIONAL HOTEL, NATIONAL HARBOR, MARYLAND**

**NOVEMBER 3–6, 2016**

The posters associated with the abstracts below are available to view at http://www.eventscribe.com/2016/posters/JADPRO/home.asp

## JL401. A Regional Cancer Center’s Experience With Lung Cancer Screening Using Low Dose CT

Aimee Strong, MSN, AGACNP-BC, Centra Medical Group, Lynchburg, VA

*Background:* Lung cancer is often diagnosed late when treatment options are limited. The goal of screening for lung cancer with low dose CT is to diagnose at an earlier stage, when more treatment options including potentially curative surgery are available. Our Regional Cancer Center partnered with Thoracic Surgery to develop a nurse practitioner led screening program to ensure that eligible patients are screened following current guidelines. The process is cumbersome for providers as the reimbursement process requires specific, time consuming documentation which may prevent providers from offering screening. There is a high rate of incidental findings which providers may be uncomfortable managing. Potential exists for patients to be lost to followup. Screening for cancer is also anxiety provoking for patients. A centralized program run by a NP with an oncology background can alleviate these issues while providing quality evidence based care. *Vision:* Advance practice providers in oncology are uniquely positioned to provide lung cancer screening. Our holistic approach to care, focusing on disease prevention and health promotion allows us to guide the patient through a potentially stressful screening process. The Thoracic NP meets with patients for the shared decision making visit and orders the CT. The NP directly follows up with patients for results, and manages all incidental findings, subsequent diagnostic procedures, referrals to specialty care while ensuring close communication with referring providers. The NP has access to weekly multidisciplinary thoracic conference to discuss significant findings and provides all longitudinal follow up regarding lung cancer screening. *Programmatic Challenges:* Lung cancer screening programs face a variety of challenges requiring collaboration of multiple departments including billing, registration, radiology, information technology, and marketing. Extensive PCP education is required on screening guidelines, including buy-in for referring patients to the screening clinic. Other challenges include complex reimbursement and documentation processes, design and implementation of patient tracking software options and education of insurance providers. Conclusion: The development and implementation of our screening program has been a valuable learning experience. To date we have screened 21 patients, one of which was found to have a squamous cell lung cancer. While our program is still new, we expect our volumes to grow over the next year as we continue to market our program in the region directly to patients and PCPs. We anticipate that our patient and provider satisfaction scores for this program will be high due to the expertise of our thoracic oncology nurse practitioner.

## JL402. A Retrospective Study of the Incidence of Vitamin D Insufficiency in BRCA Mutation Carriers in a Community Cancer Center

Phyllis C. Everett, RN, MSN, AOCN®, APNG, NP-C, Centra Medical Group, Lynchburg, VA, and Rebekah A. Betar, BS, CPHT, Liberty University, Lynchburg, VA

*Introduction:* Previous studies have alluded to the beneficial effects of vitamin D, particularly with regard to cancer. Vitamin D has been suggested to decrease negative effects of cancer treatments, hinder growth of tumor cells and reduce overall inflammation. Therefore, making sure patients have sufficient vitamin D is highly recommended. Breast cancer is something heavily researched. It is of interest to see if the development of cancerous cells is hindered by appropriate supplementation of Vitamin D. *Design:* A retrospective study to bring attention to hereditary cancer along with potential causes of the development of cancer cells associated with insufficient Vitamin D levels. *Methods:* Our preliminary study searched to determine if there was a correlation between BRCA1 and BRCA2 mutation carriers and vitamin D deficiency/insufficiency. Data was obtained from a community based oncology clinic and compared to data from a study from the same clinic in 2006. We tested 36 patients with BRCA1 and BRCA2 deleterious mutations, whereby 75% (27/36) of the patients had a Vitamin D level less than 30 ng/mL. *Results:* There was a correlation between vitamin D insufficiency with BRCA1 and BRCA2 deletions within this group of patients (n=2 deficient, n=25 insufficient, n=9 normal levels). The 9 patients with normal levels in this study averaged 38.9 ng/mL, which is significantly lower than the average literature level of 52 ng/mL (P < 0.05). Of the 20 patients with normal vitamin D levels (30-74 ng/mL), all had deletions within exonic regions. The majority of the locations of the deletions were premature truncations, where there were no intronic deletions found. *Conclusions:* We have shown a correlation of Vitamin D insufficiency and BRCA deleterious mutations. Out of the 36 patients studied with deleterious BRCA mutations, 27 of them has had insufficient vitamin D. Based on our findings, providers in our community need to be more vigilant in assessment and treatment of Vitamin D as this may impact the incidence of cancer in high risk patients. *Recommendations:* The possibility of reducing inflammation through normalizing vitamin D levels should be enough reason for primary care providers to screen everyone. There is the potential to prevent cellular damage that consequently could affect cell checkpoints, leading to the development of cancerous cells in high risk populations. Future work could include study of germline vs. somatic cells and the effect of vitamin D on the genetic integrity of the cells.

## JL403. Advanced Practice and Improved Outcomes for Infusion Therapy Patients Experiencing ADRs

Kelly R. Young, DNP, ANP-C, AOCN®, Duke Raleigh Medical Center, Raleigh, NC, Emily Dill, ANP-BC, MSN, Duke Raleigh Medical Center, Raleigh, NC, Yuri Fesko, MD, Duke Medical Oncology for Wake County, Duke University, Durham, North Carolina, and Lindsay MacDiarmada, MHA, Duke Raleigh Hospital, Raleigh, NC

*Objective:* Our cancer infusion room treats sixty patients per day. Infusions include complex chemotherapy, monoclonal antibodies, and research pharmaceuticals. The high volume of infusions and the complexity of the regimens has led to increasing numbers of adverse drug reactions (ADR) or "allergic responses". This has been primarily due to anaphylactic and anaphylactoid reactions. Most chemotherapy reactions are true allergic reactions. They occur within minutes of the start of an infusion (Lenz, 2007; Zanotti & Markman, 2001). Anaphylactoid reactions can occur within minutes to hours and are impossible to distinguish from serious reactions (Vogel, 2010). ADRs complicate 5-10% of infusions. ADRs account for increased morbidity and mortality in both chemotherapy and in monoclonal antibody infusions (Gobel, 2007). By placing an advanced practice provider (APP) in the infusion room, we anticipated better outcomes for patients experiencing ADRs. We hoped to prevent life threatening ADRs and to prevent the transfer of care to the emergency department. *Methods:* A retrospective analysis of 2016 events labeled as "allergic reactions" was completed. There were ADRs that lead to transfers to the emergency department. These ADRs were graded as a "unsafe condition" or nonevent to a "harmful" condition, or one that was temporary and required escalation in the level of care. An APP was placed in the infusion center. The APP was the "first responder" to reaction. The APP managed allergic reactions to prevent escalation of reactions and care. *Results:* The addition of the APP prevented escalating reactions that would have otherwise resulted in transfer to the emergency department. Prompt assessment and intervention led to significant decreased numbers of allergic events. While final numbers are pending, initial analysis translated into cost savings for the Center and the patients. *Conclusions:* An APP can influence the outcome of a ADR for staff and patients if available and prepared. The APP can help staff and patients navigate beyond the "standing" reaction order set when it is no longer helpful. The APP provides physical assessment skills and interventions that de-escalate the ADR both physically and fiscally. *Recommendations:* 1. APPs should routinely update themselves on the management of allergic reactions. 2. Every infusion center should be staffed with a APP who is knowledgeable about the therapies being given in the room and how to manage acute reactions. 3. APPs should be familiar with available supplies and be ready to deal with an allergic reaction from the moment an infusion is initiated.

## JL404. An Academic Cancer Center’s Approach to Observation Management Services

LaKeisha Day, PA-C, Anayo Mbadugha, PA-C, Sunil Sahai, MD, Kimberly Tripp, MBA, BSN, RN; University of Texas MD Anderson Cancer Center, Houston, TX

*Aim:* To highlight an academic cancer center’s approach to providing comprehensive care by utilizing a short-stay outpatient observation unit. *Background*: The Clinical Decision Unit (CDU) is a 16 bed observation unit that focuses on problem-based patient symptom management in collaboration with the primary oncology services. *Design:* Staffing—The CDU is staffed continuously by two Advanced Practice Providers (APPs), scheduled in alternating 12 hour shifts, with an on-call medical director for clinical and operational support. The APPs are from two existing programs that provide various inpatient and procedural services throughout the hospital. Intake/Placement—While the emergency center is the primary entry point for patients being placed in the CDU, outpatient clinics and procedural areas also request placement into the unit. The requesting service or designee makes the request for observation based on the clinical presentation at the time of the recommendation and medical necessity. Services—Once placed in the CDU, the APPs perform an initial assessment followed by patient care planning, including, a problem-focused history and physical, medication reconciliation, order management, expedition of consulting services or diagnostic exams and therapeutic procedures, where applicable. The APPs assess the patients every four to six hours to evaluate and document interim progress and make decisions on disposition. *Findings:* Placement Diagnoses—Patients placed in the CDU present with symptoms that are suitable for observation management and are at different phases of oncologic treatment. The most common diagnoses are: pain (categorized as pain of any kind), nausea/vomiting, anemia, and dehydration. Primary Service Utilization—A number of different primary oncology and surgical services populate the CDU. The top 5 services that house patients in the CDU are medical oncology services that provide treatment for solid tumor patients. Disposition Outcomes—From August 2015-June 2016, nearly 80% of patients placed in the CDU were discharged home versus those requiring an inpatient admission. Summary: Since its commencement in November 2014, the CDU has been a beneficial addition to patient care services by managing symptoms of patients who would have previously been hospitalized or forced to wait for a clinic appointment. The CDU provides dedicated, problem-focused care to patients, while emphasizing continuity of patient care by collaborating with primary oncology teams, to provide outpatient observation management services that aims to not only avoid an unnecessary hospital stay, but to also bridge any necessary care between scheduled clinic visits.

**Figure 1:**
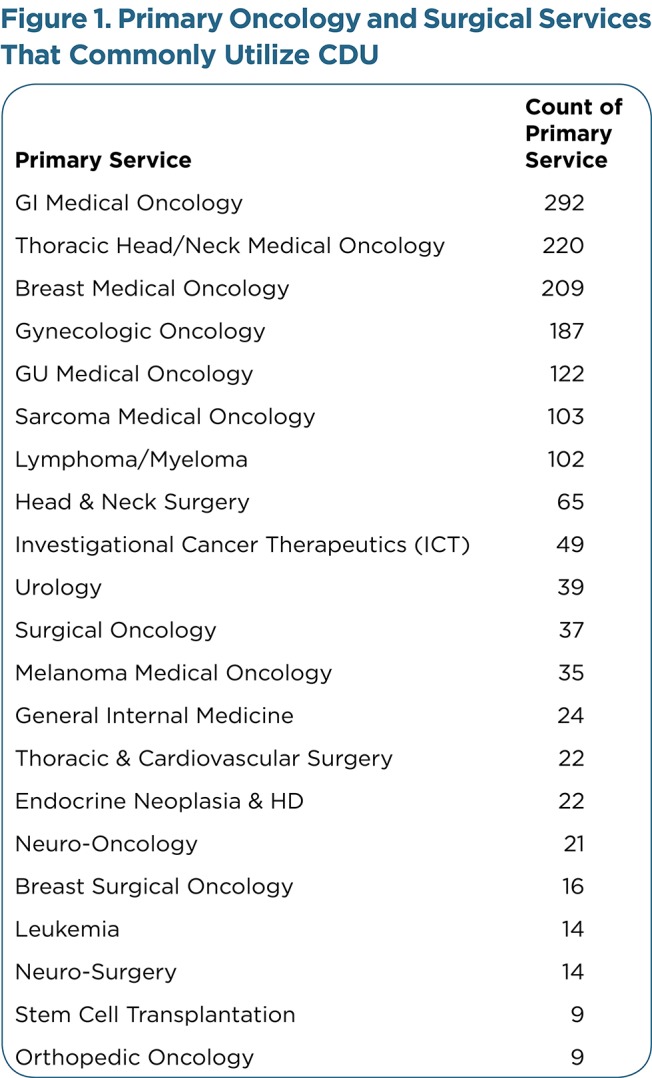
Primary Oncology and Surgical Services That Commonly Utilize CDU

**Figure 2:**
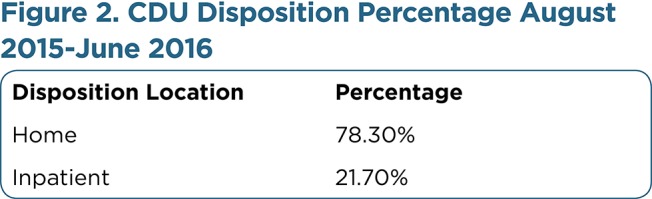
CDU Disposition Percentage August 2015-June 2016

## JL405. An Interdisciplinary Educational Checklist for Allogeneic Stem Transplant Patients

Jean A. Ridgeway, DNP, APN, NP-C, AOCN®, and Kaitlin Caponi-Holcomb, MSN, FNP-BC; University of Chicago Medical Center, Chicago, IL

*Purpose:* To develop and evaluate the use of a comprehensive educational checklist for allogeneic stem cell transplant patients, that will be used by all members of the HSCT interdisciplinary team (IDT) during initial transplant hospitalization. *Significance:* Allogeneic hematopoietic stem cell transplantation (HSCT) has emerged as a unique treatment modality. 50% of patients who received an allogeneic HSCT and caregivers report feeling inadequately equipped to manage post-discharge plan of care. HSCT patients remain one of the most fragile populations to prepare for discharge and present a challenge to be information ready. It is essential that interdisciplinary team (IDT) members prepare the HSCT patients with the education needed in order to deal with the overwhelming task involved during and after hospitalization. Healthcare checklists have produced dramatic, sustained gains in patient safety and quality of care. Checklists provide an ideal way to comply with standards of evidence based care and promote good communication among IDT members. *Sample/Methods:* IDT members of the adult HSCT program and a convenience sample of adult HSCT patients undergoing their first allogeneic stem cell transplant. The project was conducted by educating IDT members on how to use the checklist, encouraging use of the checklist as documentation of education while patients were hospitalized. Then evaluating the completed checklists items at discharge. A pilot study. *Results:* IDT educated (n=82), 22 patients consented, 20 participated (2 patients did not receive transplant). Average completion of checklist was 76%. Self-care domain (95%) was most documented domain, while adapting to post-transplant lifestyle (62%) was the least. IDT members with a nursing educational background accounted for 95% of all documentation completed. *Conclusions:* Checklists were completed by various IDT members throughout hospitalization of HSCT patients. IDT members often assume that "other" team members are completing education; however, if there is a lack of documentation there is no reliable source to verify completion of the education. Documentation was viewed as a "burden" by IDT members leading to documentation "fatigue/burnout". *Implications for Practice:* IDT checklists can only be as "interdisciplinary" as those who participate and document. Inherit care complexity of patients with multiple "teams", can be streamlined with a checklist. This checklist allows for the IDT members to engage in meaningful discussions with patients and caregivers about their plan of care. From this discussion, that goes beyond the standardized education of HSCT patients, allows IDT members to develop a plan to effectively manage their care following discharge.

**Figure 3:**
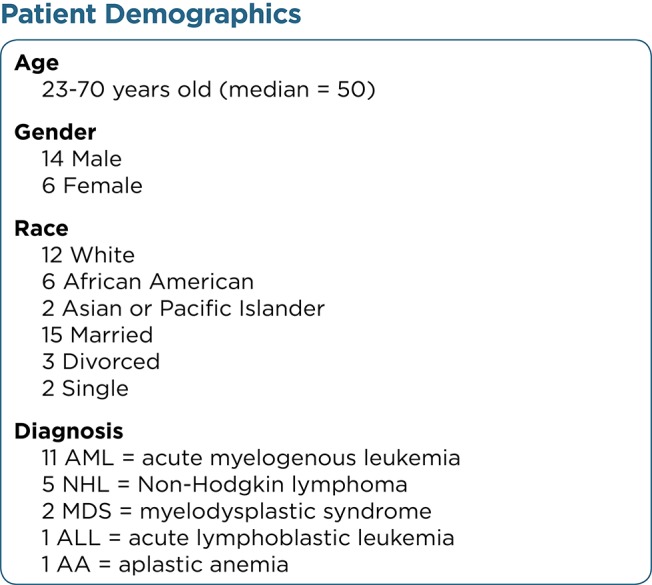
Patient Demographics

## JL406. BMDACC Breast Cancer Program Clinical Rotation for Nurse Practitioner Students

Michele Sazama, DNP, ANP-C, Melissa Shelby, MSN, ACNP-BC, RNFA, Aida Amado, MSN, ACNP-BC, Stefanie Casey, MSN, ACNP-BC, and Lynn Schuster, DNP, ACNP-BC; Banner MD Anderson Cancer Center, Gilbert, AZ

*Purpose:* To increase awareness and promote understanding of multidisciplinary care for breast cancer patients. Background: Clinical rotations are designed to bridge the gap between didactic and practice. However, there are significant variances in the quality of clinical rotations provided for nurse practitioner students, particularly in specialty areas like oncology. It is becoming increasingly difficult for graduate students to find supportive clinical mentors. *Design:* Creation of a formal 80-hour clinical rotation for adult, acute care, and doctoral nurse practitioner students. Students work directly with nurse practitioner mentors who utilize holistic evidence-based practice techniques. *Methods:* Students are given a pre-test at the beginning of the clinical rotation, then provided evidence-based articles to read throughout their time on campus. They are given a detailed itinerary, and specific clinical objectives. Students rotate through surgical oncology, medical oncology, radiation oncology, integrative medicine, and specialty services. *Findings:* Students completed a post-test at the end of the clinical rotation. At the end of one year (May 2015 to May 2016), program information was analyzed. All scores increased (n=8), with the average of 43.21% improvement. Clinical mentors provided a written student evaluation based on both unique program and NONPF nurse practitioner core competencies. Students provided positive verbal and written feedback regarding the clinical rotation. *Conclusions:* Creation of a multidisciplinary formal clinical rotation for nurse practitioner students provided multiple benefits, including: academic leadership, promotion of facility awareness at the state and national level, quality improvement project for NAPBC accreditation, advanced practice provider recruitment, and nurse practitioner retention. All students demonstrated an improved knowledge of oncology and an increased proficiency with their clinical skill set. *Implications:* We were able to establish and build positive relationships with nurse practitioner program directors and deans at the major state universities, and obtained adjunct faculty status for all members of our mentor team. For the second year of our program, we expanded eligibility by offering our program to women’s health and family nurse practitioner students. *Clinical Relevance:* Cancer is the second leading cause of death in the United States (CDC, 2015). It is critical that nurse practitioners have the skill set to evaluate diagnostics and pathology reports, and expedite referral to oncology specialists to improve patient outcomes. This project demonstrates the ability of experienced nurse practitioners to positively impact the next generation of oncology practitioners.

## JL407. Burden of Phlebotomy in Patients With Polycythemia Vera in the United States: A Preliminary Analysis of Baseline Data from the REVEAL Study

Brady L. Stein, MD, MHS, Northwestern University Feinberg School of Medicine, Chicago, IL, Ralph V. Boccia, MD, FACP, The Center for Cancer and Blood Disorders, Bethesda, MD, Alison R. Moliterno, MD, Johns Hopkins University School of Medicine, Baltimore, MD, Michael R. Grunwald, MD, Levine Cancer Institute, Carolinas HealthCare System, Charlotte, NC, Stephen T. Oh, MD, PhD, Washington University School of Medicine, St. Louis, MO, Ahmad B. Naim, MD, Incyte Corporation, Wilmington, DE, Dilan C. Paranagama, PhD, Incyte Corporation, Wilmington, DE, Hao Sun, MS, Incyte Corporation, Wilmington, DE, Shreekant V. Parasuraman, BPharm, PhD, Incyte Corporation, Wilmington, DE, Ruben A. Mesa, MD, FACP, Mayo Clinic Cancer Center, Scottsdale, AZ

*Background:* Phlebotomy is often used to maintain hematocrit < 45% and prevent thrombotic events in patients with polycythemia vera (PV); however, phlebotomy procedures are inconvenient and/or poorly tolerated by some patients. Iron deficiency resulting from phlebotomy may be associated with burdensome symptoms, including fatigue, impaired cognitive function, and restless legs syndrome. The ongoing REVEAL study (NCT02252159) aims to describe contemporary demographics, disease burden, clinical management, and patient-reported outcomes among US patients with PV. This analysis presents the patient-reported burden of phlebotomy for currently enrolled patients (data cutoff: April 28, 2016). *Methods:* REVEAL is a prospective, observational study enrolling adults aged ≥18 years with PV under physician supervision. Physician assessments and patient-reported outcomes are being collected during a 36-month period. Phlebotomy-related burden in patients and their caregivers is evaluated using the 21-item phlebotomy burden questionnaire (PBQ-21), assessing phlebotomy frequency, time required for phlebotomy, practice setting, inconvenience, and phlebotomy-related adverse effects. Phlebotomy-related effects and bother/inconvenience (≤24 hours following the procedure) are graded from 1 (not at all) to 4 (extremely). *Results:* At data cutoff, 2307 patients were enrolled (planned enrollment, n=2500); 1105 patients completed PBQ-21 and had a phlebotomy within the previous 3 months. Median (range) age was 65 (22–95) years; the majority were male (61.5%) and white (91.9%). Median disease duration (PV diagnosis to enrollment) was 3.3 years. Mean (SD) number of phlebotomies was 2.2 (1.6), and total treatment time was 4.2 (5.1) hours per procedure. Employed patients (n=488) reported missing 4.9 (13.3) hours of work time per procedure. Phlebotomy was most often performed at an infusion or specialized cancer care center (32.5%) or at a physician’s office during a regular medical visit (30.0%). Most patients (72.5%) experienced ≥1 phlebotomy-related adverse effect(s); the most common are reported in Table 1. Many patients reported that phlebotomy procedures were moderately or extremely bothersome (18.0%), inconvenient (15.9%), painful or physically uncomfortable (16.1%), and inconvenient for family and friends (8.2%). *Conclusions:* PBQ-21 is a novel and valuable instrument to assess the effects of phlebotomy on patients and caregivers. Fatigue, bruising, dehydration, and dizziness resulting from phlebotomy procedures were reported in a considerable proportion of patients with PV. Additionally, these preliminary data suggest that phlebotomy treatment is associated with pain, discomfort, inconvenience, and notable time commitments in some patients. *Implications:* These findings provide physicians, patients, and caregivers with important insight into the burden of phlebotomy in patients with PV and may help guide individualized treatment decisions.

**Table 1:**
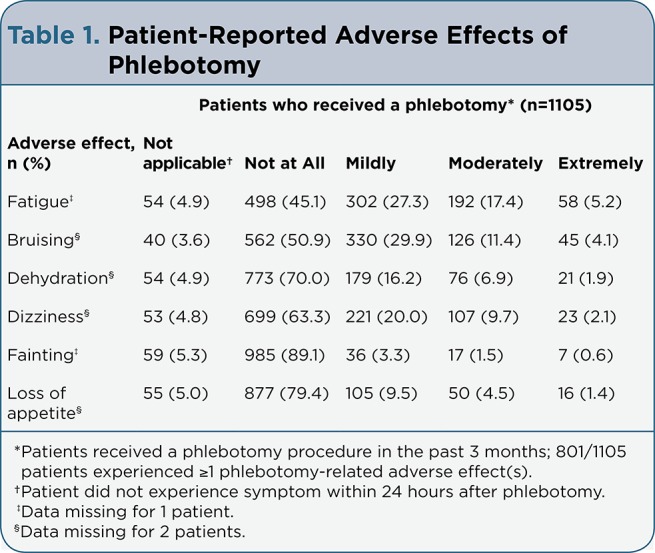
Patient-Reported Adverse Effects of Phlebotomy

## JL408. Decreasing Venous Thromboembolism in Patients With Hematologic Malignancy Through Implementation of Clinical Practice Guidelines – A Quality Improvement Project

Karley M. Trautman, DNP, ANP-BC, and Chelsey Boggs, MS, AGNP-BC; Blood Cancer and Bone Marrow Transplant Program, University of Colorado Anschutz Medical Campus, Aurora, CO

*Background:* Venous thromboembolism (VTE) is a highly prevalent complication in patients with cancer with reported incidence rates ranging between 8-19% (Khorana, Dalal, Lin, & Connolly, 2013). Patients with VTE have at least a 2-fold increase in mortality compared to those without VTE, even after adjusting for staging (Khorana & Connolly, 2009). Because of this, there is a justifiable need to decrease the incidence of VTE by standardizing practice to align with the current evidence-based practice (EBP) guidelines while simultaneously not increasing bleeding risk in patients with thrombocytopenia. *Objective*: The aim was to decrease the rate of acute VTE and recurrent VTE in adults with hematologic malignancies by standardizing the risk assessment, use of prophylactic anticoagulation, and treatment duration of therapeutic anticoagulation without increasing bleeding risk in the inpatient and outpatient clinical settings at the University of Colorado Blood Cancers and Bone Marrow Transplant Program. *Intervention/Methods:* The National Comprehensive Cancer Network (NCCN) VTE guidelines were used to develop a clinical practice guideline addressing standard VTE risk assessment, appropriate use of prophylactic anticoagulation, and standardized duration of therapeutic anticoagulation. Data was collected for 12 months prior to the implementation of the guideline and for 24 month post-implementation. *Results:* Overall VTE rates decreased from 13.99% to 6.18%, recurrence rate decreased from 42.5% to 20.93%, and bleeding risk improved from 18.52% to 7.06%. Percentage of inpatients receiving prophylactic anticoagulation increased from 25.2% to 42.58% and eligible outpatient myeloma patients receiving prophylaxis increased from 82.76% to 96.97%. *Conclusions:* Standardization and development of clinical practice guidelines improved VTE incidence and recurrence rates and improved overall adherence to EBP guidelines. Routine prophylactic anticoagulation did not increase bleeding risk in this patient population. *Implications for Practice:* EBP guidelines aid providers in making sound clinical decisions regarding the management of VTE in patients with hematologic malignancies. Quality improvement projects have a vital role in effective translation of EBP in practice. Data updated at time of publication of this JADPRO issue

## JL409. Development of An Evidence-Based Scorecard to Assess Provider Adherence to Gastrointestinal Toxicity Management

Carrie T. Stricker, PhD, RN, Carevive Systems, Inc., Miami, FL, Debra Wujcik, PhD, RN, FAAN, Carevive Systems, Inc., Miami, FL, June Eilers, PhD, APRN-CNS, BC, FAAN, University of Nebraska Medical Center, Omaha Division, Omaha, NE, Beth Faiman, PHD, MSN, APRN-BC, AOCN®, Cleveland Clinic, Cleveland, OH, Amy Goodrich, CRNP, Johns Hopkins Kimmel Cancer Center, Baltimore, Maryland, Mary Pat Lynch, MSN, CRNP, AOCNP®, Abramson Cancer Center, Hospital of the University of Pennsylvania, Beth Eaby-Sandy, MSN, CRNP, OCN®, Abramson Cancer Center, Hospital of the University of Pennsylvania, William Dudley, PhD, UNC Greensboro, Greensboro, NC, Susan L. Beck, PhD, APRN, FAAN, University of Utah College of Nursing, Salt Lake City, Utah, Karen J. Hammelef, DNP, RN, Carevive Systems, Inc., Miami, FL

*Objective:* Gastrointestinal toxicity (GIT) is associated with targeted therapies in cancer treatment. GIT can lead to diminished treatment adherence and poor quality of life. It is among the top causes of emergency room visits and hospitalizations –targeted by value-based payment models including the Oncology Care Model (OCM). Evidence for the prevention and management of GIT exists. Yet, what constitutes "good" versus "poor" GIT management (GITM) has not been defined in a measureable way; little is known about how evidence based guidelines are applied in the real-world practice environment. Because patients on oral oncolytics are typically seen less frequently in the clinic, health care providers are not routinely observing patients. The processes for patient reporting of symptoms and clinician triaging, evaluating, and managing GIT are inconsistently documented in the patient’s health record. Thus, the effect that real world GITM practices have on patient outcomes is unknown. This formative work is the initial part of a larger study to understand real world GITM by defining quality management. Next, the study will quantify provider adherence to these quality management standards. *Methods:* An expert oncology nurse researcher led a team of 5 oncology advanced practice nurses (OAPN) in the development of an instrument to define quality GITM. The GITM ScoreCard (SC) was developed through processes that included literature review and consensus development for ideal management where evidence gaps existed. The OAPNs developed literature review results of GITM evidence-based practices using a standardized template. The nurse researcher reviewed the literature review results for consistency, flagging sections requiring further discussion. Consensus was achieved through two scheduled sessions with the entire group. *Results:* An evidence-based SC was developed that can be used in any clinic setting to assess GITM practices. The SC includes four symptoms: Diarrhea, Constipation, Mucositis, and Nausea/Vomiting. GITM practices include 1) prevention, 2) assessment 3) pharmacologic and non-pharmacologic management by toxicity grade, and 4) other. Scoring for each response is 1) Never, 2) Occasionally, < than half time, 3) Most of the time, > half time, and 4) Always. Conclusion: Development of evidence-based GITM SC is the first step in understanding real world GITM. Provider self-assessment using the SC will provide the first known documentation of provider adherence to evidence-based GITM. *Recommendations:* The GITM SC has the potential to be used in any clinic setting that desires to evaluate and improve the quality of their current GITM practices.

## JL410. Identification of Continued Educational Gaps Among Oncology Nurses Treating Patients With Pancreatic Cancer: A Case for Further Education

Nina N. Grenon, DNP, AOCN®, Dana-Farber Cancer Institute, Boston, MA, and Patricia M. Repetto, M.Ed, CHCP, Medscape Oncology, New York, NY

*Introduction/Background:* In recent years, the treatment paradigm for management of advanced pancreatic cancer has changed dramatically. Because oncology nurses are on the front line of care for patients with pancreatic cancer, we sought to evaluate their knowledge and competence in the care of patients with advanced disease and the impact of case-based education on narrowing gaps in clinical practice. *Materials and Methods:* An online educational program designed as a video roundtable discussion between nurses and tailored to a nursing audience was posted on Medscape Oncology January 13, 2016, and ran until March 8, 2016 (http://www.medscape.org/viewarticle/855566). Educational efficacy was determined by using each learner as their own control and comparing each participant’s responses to a series of 4 questions posed before exposure to educational content (pre-assessment) with his/her responses the identical questions posed after exposure to the educational content (post-assessment). Responses of participants who answered all pre- and post-assessment questions during the study period were included in the analysis. McNemar’s chi-square test was used to assess differences from pre- to post-assessment responses for all questions combined. Cramer’s V was used to estimate the strength of the association between pre- and post-assessment test scores. P values are shown as a measure of significance with P<.05 indicated statistical significance. *Results:* Nearly 13,000 nurses participated in the activity, of which 1896 qualified for inclusion in this study. Participation in this educational program resulted in a medium effect size (Cramer’s V = 0.189; P < .05). Significant improvements included (Figure 1; all P < .05):

- A relative 81% improvement in knowledge related to the management of pancreatic insufficiency in patients (P < .05)
- A relative 46% improvement in comprehension of the role palliative care has in the management of patients with advanced pancreatic cancer (P < .05), although nearly 30% still selected incorrect responses after the activity
- A relative 31% improvement in recognition that one of the most common treatment-related adverse effects of nanoliposomal irinotecan is neutropenia (P < .05)
- A relative 36% improvement in distinguishing differences in the adverse effect profiles of nab-paclitaxel with gemcitabine versus gemcitabine monotherapy (P < .05)

*Conclusions:* This study identified ongoing educational gaps that suggest development of additional education is needed to improve oncology nurse knowledge and competence in the management of pancreatic cancer, especially in recognition of the value of supportive and palliative care as well as recognition of distinguishing characteristics of the adverse effect profiles of evidence-based regimens.

## JL411. Implementation and Evaluation of an Influenza Adherence Toolkit in Allogeneic Bone Marrow Transplant Recipients

Tracy Krimmel, DNP, Rutgers Cancer Institute of New Jersey, New Brunswick, NJ, Susan M. Schneider, PhD, RN, AOCN®, FAAN, Duke University School of Nursing, Durham, NC, Rajat Bannerji, MD, PhD, Rutgers Cancer Institute of New Jersey, New Brunswick, NJ, and Maribel Borysyuk, PharmD, Walgreens, Dallas, TX

*Background:* The incidence of influenza in hematopoietic stem cell recipients is 7-35% with a 15-35% mortality rate (Shah et al., 2012). Vinograd et al. (2014) reported a 48% compliance rate in influenza vaccinations. In our NCI-designated cancer center, the baseline adherence rate with influenza vaccination in the post-allogeneic setting was 62.5%. *Methods:* This project was a pre-test post-test quality improvement project to determine patient knowledge and vaccination adherence rates following implementation of the influenza adherence toolkit. The intervention included: 1) Pre and Post screening survey, 2) The toolkit which includes an influenza disease education pamphlet and financial incentive, and 3) reminder letter. *Results:* There were 48 eligible patients for the project, 32 completed the pre-screening questionnaire (66.7%). The results showed overall adherence rate for 2015-2016 to be 88.5%, compared to a baseline vaccination rate of 62.5%. Findings also revealed a strong association between provider recommendations and influenza vaccination adherence, 23 of the 24 (96%) patients who had a provider recommend receiving the flu shot did receive it (p=.009). Conclusion: Findings showed that implementation and evaluation of an influenza toolkit in allogeneic hematopoietic recipients improves adherence rates. Consistent with previous studies, there was a strong association between provider recommendation and obtaining the vaccination.

## JL412. Management of Endocrinopathies Associated With Nivolumab and Ipilimumab Combination Therapy in Solid Tumors

Marianne Davies, DNP, ACNP, AOCNP®, Yale University School of Medicine, New Haven, CT, Laura S. Wood, RN, MSN, OCN®, Cleveland Clinic, Cleveland, OH, Krista Rubin, MS, FNP-BC, Massachusetts General Hospital, Boston, MA, Kathleen Madden, NP, MSN, FNP-BC, AOCNP®, APHN, New York University, New York, NY, Laura Brennan, NP, AOCNP®, UC Davis Medical Center, Sacramento, CA, Nathan Dahl, PharmD, RPh, Mayo Clinic, Rochester, MN, Dana Walker, MD, MSCE, Bristol-Myers Squibb, Princeton, NJ, Paul Gagnier, PhD, MD, Bristol-Myers Squibb, Princeton, NJ, Xuemei Li, MD, Bristol-Myers Squibb, Princeton, NJ, and Lisa Kottschade, APRN, MSN, CNP, Mayo Clinic, Rochester, MN

*Background:* Combination therapy with nivolumab (NIVO) and ipilimumab (IPI) is approved for the first-line treatment of advanced melanoma (MEL). Recent phase 1 studies have also shown promising efficacy with NIVO+IPI in patients (pts) with non-small cell lung cancer (NSCLC) and metastatic renal cell carcinoma (RCC). With NIVO+IPI, select adverse events (ie, immune-related AEs) most commonly affect the skin, gastrointestinal tract, endocrine organs, and liver. Unlike other immune-related AEs, endocrinopathies can persist despite discontinuation or completion of therapy. Here, we review endocrine select AEs associated with NIVO+IPI in solid tumors and provide practical guidance regarding their management. *Methods:* Safety data were included from phase 2 (CheckMate 069) and phase 3 (CheckMate 067) studies for MEL, and phase 1 studies for NSCLC (CheckMate 012) and RCC (CheckMate 016). Data are reported for NIVO 1 mg/kg + IPI 3 mg/kg Q3W x 4 for MEL, NIVO 3 mg/kg Q2W + IPI 1 mg/kg Q6W for NSCLC, and NIVO 3 + IPI 1 or NIVO 1 + IPI 3 mg/kg Q3W x 4 for RCC, followed by NIVO Q2W. Pts were treated until disease progression, unacceptable toxicity, or withdrawal of consent. *Results:* In pts with MEL who received NIVO+IPI combination therapy, ~32% experienced any endocrine select AE, of which 5-6% were grade 3-4 (Table). Endocrine select AEs of any grade occurred in 21% of pts with NSCLC, in 28% of pts with RCC who received NIVO 3 + IPI 1 mg/kg, and in 43% of pts with RCC who received NIVO 1 + IPI 3 mg/kg. The most common treatment-related endocrine select AEs across tumor types were hypothyroidism, hyperthyroidism, and adrenal insufficiency. Median time to onset for endocrine select AEs was 6 weeks in MEL. Only 2-3% of pts discontinued due to treatment-related endocrine select AEs. Some pts (typically those with lower grade AEs) can continue NIVO if the AEs were related to IPI. Persistent or worsening fatigue, headaches, and nausea are common symptoms, and may be associated with hypophysitis. Baseline labs (including thyroid-stimulating hormone) should be performed. MRI with pituitary cut can be used to evaluate potential immune-mediated hypophysitis. Pts with thyroid dysfunction need symptomatic management and thyroid hormone replacement, and those with adrenal insufficiency may require long-term glucocorticoid replacement. *Implications:* Endocrine select AEs are manageable with established treatment algorithms. Given the unique nature of endocrine AEs, early identification is critical and a multidisciplinary team approach is required to effectively manage these pts.

**Table:**
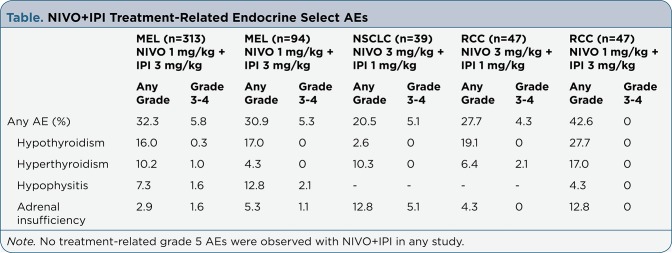
NIVO+IPI Treatment-Related Endocrine Select AEs

## JL413. Management of Gastrointestinal Adverse Events (AEs) Associated With Nivolumab and Ipilimumab Combination Therapy in Solid Tumors

Lisa Kottschade, APRN, MSN, CNP, Mayo Clinic, Rochester, MN, Marianne Davies, DNP, ACNP, AOCNP®, Yale University School of Medicine, New Haven, CT, Krista Rubin, MS, FNP-BC, Massachusetts General Hospital, Boston, MA, Kathleen Madden, NP, MSN, FNP-BC, AOCNP®, APHN, New York University, New York, NY, Nathan Dahl, PharmD, RPh, Mayo Clinic, Rochester, MN, Laura Brennan, NP, AOCNP®, UC Davis Medical Center, Sacramento, CA, Dana Walker, MD, MSCE, Bristol-Myers Squibb, Princeton, NJ, Xuemei Li, MD, Bristol-Myers Squibb, Princeton, NJ, Paul Gagnier, PhD, MD, Bristol-Myers Squibb, Princeton, NJ, and Laura S. Wood, RN, MSN, OCN®, Cleveland Clinic, Cleveland, OH

*Background:* Nivolumab (NIVO) and ipilimumab (IPI) combination therapy is approved for the first-line treatment of advanced melanoma (MEL), and recent phase 1 studies have shown promising efficacy in non-small cell lung cancer (NSCLC) and metastatic renal cell carcinoma (RCC). NIVO+IPI is associated with a higher frequency of treatment-related AEs compared to monotherapy, with select AEs (ie, those with an immune-related etiology) in the skin and gastrointestinal (GI) tract being the most common. Here, we review the incidence of GI select AEs in patients (pts) treated with NIVO+IPI and present practical guidance regarding their management. *Methods:* Safety data were included from phase 2 (CheckMate 069) and phase 3 (CheckMate 067) studies for MEL, and phase 1 studies for NSCLC (CheckMate 012) and RCC (CheckMate 016). Data are reported for NIVO 1 mg/kg + IPI 3 mg/kg Q3W x 4 for MEL, NIVO 3 mg/kg Q2W + IPI 1 mg/kg Q6W for NSCLC, and NIVO 3 + IPI 1 or NIVO 1 + IPI 3 mg/kg Q3W x 4 for RCC, followed by NIVO Q2W. Pts were treated until disease progression, unacceptable toxicity, or withdrawal of consent. *Results:* In MEL pts treated with NIVO+IPI, ~48% experienced any grade GI select AEs; 15-20% experienced grade 3-4 AEs, with the most common being diarrhea and colitis (Tabl). Any grade GI select AEs occurred in 23% of pts with NSCLC, in 26% of pts with RCC who received NIVO 3 + IPI 1 mg/kg, and in 45% of pts with RCC who received NIVO 1 + IPI 3 mg/kg. To effectively manage GI AEs, pts need to report all GI symptoms (e.g., increased stool frequency, loose consistency, abdominal cramping, and bloody diarrhea) promptly to their oncology healthcare provider. Pts with diarrhea should undergo stool testing to rule out infectious causes. GI AEs can progress rapidly, and thus healthcare providers should initiate treatment at the first sign of symptoms (corticosteroids for pts with persistent grade ≥2 AEs) even before stool testing results are available, in order to prevent serious complications of intestinal inflammation such as bowel perforation. *Implications:* Across solid tumor types, GI select AEs are among the most commonly reported side effects in pts who receive NIVO+IPI therapy. With early recognition and intervention, serious complications from GI select AEs may be minimized using established management guidelines involving immune modulating medications. Pt education and frequent follow-up are also key to minimizing serious complications from these AEs.

**Table:**
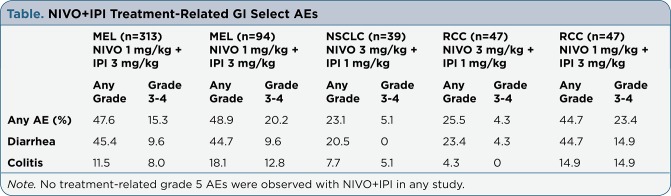
NIVO+IPI Treatment-Related GI Select AEs

## JL414. Multidisciplinary Management of Hepatocellular Carcinoma: The Role of Systemic Chemotherapy and Chemoembolization in Converting Patients With Large Unresectable Liver Tumors to Surgical Resectability

Jenilette D. Velasco, MPAS, PA-C, and Steven H. Wei, MS, MPH, PA-C; MD Anderson Cancer Center, Houston, TX

*Introduction:* HCC is the fifth most common malignant neoplasm in men and ninth most common in women worldwide. Although the incidence of HCC in the United States is rare, the incidence of this particular cancer is rising secondary to the increasing prevalence of hepatitis B, hepatitis C, alcohol abuse, and non-alcoholic steatohepatitis (NASH). In the setting of liver transplantation exclusion, surgical resection remains the gold standard for patients with resectable HCC. However, most patients with HCC present with unresectable tumors in the background of chronic liver disease. Furthermore, the underlying liver disease, such as cirrhosis, is a major confounding factor that contributes to its chemotherapy-resistant characteristic. In patients with unresectable HCC in the background of a normal liver, however, systemic chemotherapy has been shown to be somewhat effective, but its role in the neoadjuvant setting remains controversial. Additionally, transarterial chemoembolization (TACE) is an effective locoregional approach used to downsize tumors prior to surgical resection. Can the combination of systemic chemotherapy and TACE be utilized in the neoadjuvant setting to potentially preserve the surgical option for this patient population? We present a case study that details the unique role of systemic chemotherapy and TACE in downstaging patients with large and unresectable HCC in the background of a normal liver to resectability. *Description:* Case: 60-year-old gentleman with a solitary 17 cm left liver mass, encroaching the right liver with displacement of the main portal vein and involvement of the right hepatic vein, biopsy-proven HCC. He had no risk factors for HCC (negative for hepatitis B & C; nondrinker). The patient was deemed unresectable at that time and was dispositioned to systemic chemotherapy and TACE with the goal of down-sizing the tumor for surgical resection with good margins. Conclusion: In patients with initially unresectable and large HCC in the background of a normal liver, the role of systemic chemotherapy and TACE should be considered as a neoadjuvant treatment modality to convert the patient for surgical resection. Advanced practice providers play a vital role in the process of the treatment planning for each patient. We hope to provide an alternative and effective treatment plan for patients with HCC in the background of normal liver while preserving the surgical option to patients who were deemed initially unresectable.

## JL415. Nurses Championing Exercise in Survivorship Care Clinic Visits

Bernadette Labriola, MSN, FNP-C, Duke University, Durham, NC, and Kathy Trotter, DNP, FAANP, Duke School of Nursing, Durham, NC

*Objective:* Breast cancer survivors are highly motivated to make lifestyle changes that enhance health (Urquhart, 2010). Nurses and nurse practitioners (NPs) often provide cancer survivor care and are in an influential position to educate on the importance of starting or maintaining moderate exercise activities. These may include walking, strength training, aerobic activity, and dance. Specifically, the ONS campaign Get Up and Get Moving campaign supports evidence based practice for oncology nurses to provide individualized activity recommendations to a goal of 100,000 patients in 2016. Nurses can support exercise benefits with encouragement that exercise is a great "medicine" (or therapy?) for the entire mind and body. Physical activity has been shown to improve post cancer treatment related symptoms such as fatigue, muscular strength, aerobic fitness, and quality of life (Baruth, 2013; Hayes, 2013; Schmitz, 2010; Wenzel, 2013; Mayo, 2014). Exercise also decreases the risk of cancer recurrence (Siegel, 2012). *Methods:* A retrospective chart review (n=25) was accomplished from 1/2015 to 7/2016 in an outpatient breast cancer clinic in a comprehensive cancer center. All chart visits were follow up surveillance visits by a solo NP. Metric 1: Charts were analyzed for an exercise history at each clinic visit with documentation of activity, frequency, and barriers. Metric 2: Charts were analyzed for documentation of patient counseling about an exercise plan with type/frequency/length of time. *Results:* Metric 1: Documentation of exercise query for 22/25 patients = 78% compliance. Metric 2: Exercise prescription documented for 12/25 patients = 52% compliance. *Conclusions:* Nurses and nurse practitioners can and do counsel and document exercise levels. *Recommendations:* More quality assurance research is needed to determine optimum methods of follow up and reinforcement for continued exercise adherence. Implementation of this process could be improved by streamlining history taking during routine visits with the use of a physical activity tool (Godin, 2011) and a standard phrase in the EMR survivor care template. Time constraints in a busy outpatient oncology clinic can be addressed by incorporating the use of assistive personnel distributing the exercise assessment tool, reinforcing teaching and assisting with follow up phone calls. Further, resources to consider may be an exercise physiologist, YMCA Life for Life programs, Institutional walking survivor groups.

## JL416. Parathyroid Carcinoma Overview: A Case Study

Elizabeth Sanders-Schacht, PA-C, and Rae Brana Reynolds, MS, RN, ANP; MD Anderson Cancer Center, Houston, TX

*Introduction:* Parathyroid carcinoma is a rare malignancy that can be difficult to diagnose due to clinical features shared with benign primary hyperparathyroidism (PHPT). It accounts for 1% of PHPT cases and has symptoms commonly referred to as "bones, stones, abdominal moans, and psychic groans," attributable to hypercalcemia, elevated parathyroid hormone (PTH), nephrolithiasis, cognitive dysfunction, insomnia, fatigue, and bone demineralization. Compared with benign PHPT, parathyroid carcinoma can present with more pronounced elevation of calcium and PTH, as well as a palpable neck mass that is rare in benign HPT. Complete, negative-margin resection is the standard of care. Because of its rarity, there is currently no consensus on the Tumor, Node, Metastasis (TMN) staging of parathyroid carcinoma. *Purpose:* The purpose of this presentation is to update Advanced Practice Providers (APPs) on the diagnosis, evaluation, management, and oncologic surveillance of parathyroid carcinoma. *Discussion:* Case: A 48 year old woman presents with fatigue, constipation, and bone pain. Evaluation revealed a serum calcium of 14.2, prompting a PTH measurement which was elevated at 312. Localization of a presumed parathyroid adenoma was completed. Sestamibi scan and ultrasound of the neck revealed a large left inferior parathyroid adenoma. The patient underwent a parathyroidectomy with an en bloc resection left lobectomy, excision of sternocleidomastoid muscle, and a few surrounding lymph nodes. Pathology revealed parathyroid carcinoma. Given the malignancy findings, a staging CT scan followed which did not reveal metastasis. She was evaluated every 6 months with a calcium and PTH level. Her levels remained normal until 2 years post-operatively when her calcium was elevated at 11.9 and subsequent Neck CT revealed cervical adenopathy. She was then referred to a high-volume cancer center where biopsy confirmed recurrent disease in the left neck and superior mediastinum, and she underwent lymphadenectomy. Unfortunately, she later presented with metastasis to the liver. This case will be presented to elucidate the unique features of parathyroid carcinoma along with its diagnosis and management. Conclusion: Parathyroid carcinoma is rare, but it is vital for APPs to be knowledgeable of its appropriate evaluation and management as recurrence and persistent disease can be a problem in more than 50% of patients. It is important to be well-informed in order to manage, counsel, and refer patients appropriately. Much work is needed towards formulating a TMN staging system to guide treatment. Accurate diagnosis, pathological evaluation, and surgical documentation is vital to long term development of a staging system.

## JL417. Performance Improvement Continuing Medical Education (CME) for the Advanced Practice Provider in Oncology

Kelly R. Maldonado, MPAS, PA-C, Maura Polansky, MS, MHPE, PA-C, Jessica Eno, MS, PA-C, Leah Theriot, MPAS, PA-C, Carolyn Zawislak, MPAS, PA-C, and Steven H. Wei, MS, MPH, PA-C; University of Texas MD Anderson Cancer Center, Houston, TX

*Background:* Performance improvement (PI) is one of the newest CME learning formats designed for providers to evaluate and make changes to their clinical practice. In 2014, The National Commission on Certification of Physician Assistants (NCCPA) implemented a change in the recertification process to include PI as part of the required CME category 1 credits. Following the end of a ten-year cycle, physician assistants must have at least 40 category 1 PI-CME credits. PI-CME is different from other category 1 activities as it involves a structured and systematic three-stage approach by which evidenced based performance measures are used to help providers identify areas of improvement in their clinical practice. In this three-stage process, the provider must first assess their practice by comparing it directly to an evidence based performance measure. The provider then must analyze and consider the results of the data for areas of improvement and identify one or more strategies for improvement. After implementing a strategy and collecting additional data following the implementation, the provider must finally reflect on the results. The advantage of PI-CME is that it can be developed for any subspecialty of practice. It can essentially address any process related to health care delivery. *Purpose:* To update physician assistants of the PI-CME requirements for certification maintenance and to provide advanced practitioners an overview of the stages of developing a PI-CME activity as it relates to improving patient care in the oncology practice setting. *Description:* We describe 3 PI CME activities developed by physician assistants at the University of Texas MD Anderson Cancer Center to be used by physician assistants in the fields of medical, surgical, and radiation oncology. These activities were developed by and for physician assistants to address specific patient care issues related to the care of the oncology patient such as accuracy in communicating patient hand offs, proper monitoring of patients receiving chemotherapy, and ensuring appropriate follow up evaluation after completion of radiation therapy. Conclusion: PI-CME is a structured tool that is relevant to all advanced practice providers, not only physician assistants, and is an active application of learning which ultimately leads to improved patient outcomes. PI-CME is applicable to any subspecialty of practice, including the specialized fields of oncology.

## JL418. Primary Polycythemia Vera: Advances in Diagnosis and Treatment

Kathy Leonard, MA, ACNP-B, ANP-B, ANP-BC, AOCNP®, New York University Clinical Cancer Center, New York, NY

*Purpose:* The purpose of this poster presentation is to educate advanced hematology/oncology practitioners how to diagnose, evaluate thrombotic risk, manage and treat patients with primary polycythemia vera. *Background*: Primary polycythemia vera is a clonal stem cell disorder characterized by erythrocytosis, as well as leukocytosis and thrombocytosis. It is a Philadelphia chromosome negative subtype of the myeloproliferative neoplasms. The age of diagnosis is usually in the sixth decade and is rarely seen in individuals under the age of thirty. The condition was first described in the late 1800s, and it wasn’t until 2005 that a mutation of the JAK2 (Janus kinase 2) gene was identified as playing a role in this disease. Polycythemia vera may result in life threatening thrombotic events, progression to myelofibrosis and acute myeloid leukemia. The primary treatment is aspirin and therapeutic phlebotomy with platelet lowering in select patient populations. The identification of the JAK 2 V617F mutation has led to a more definitive diagnosis and has made way for the use of JAK inhibitors. *Discussion:* Treatment of polycythemia vera is aimed at prevention of life threatening thromboembolic events through careful risk stratification, identification and monitoring of side effects of pharmacological agents and early identification of disease progression to myelofibrosis and acute myeloid leukemia. *Implications for Advanced Practice:* It is very important that advanced hematology/oncology practitioners have a strong knowledge and understanding of the diagnosis, management and treatment of this disease. The advanced practitioner has a pivotal role in treatment and management of those with polycythemia vera.

## JL419. Recognizing the Contributions of Advanced Practice Providers to Oncology Care: Are Current Metrics Enough?

Amanda W. Yopp, MSN, AGNP-BC, Holly Wall, RN, MSN, ACNP-BC, and Kena Miller, RN, MSN, ARNP-BC; Takeda Oncology, Cambridge, MA

*Background:* The contributions of advance practice providers (APPs) to an oncology practice are complex, and it can be challenging to discern and measure how their unique contributions are impacting their practice. Two terms, productivity and value, are used to measure contributions. APPs should have a clear understanding of their practice’s definition of these terms and their expectations for evaluation of APP contributions, because when APPs’ contributions are valued, their job satisfaction and team productivity are likely to improve. In addition, proper documentation of the quality and cost-effectiveness of the care provided by the APP is crucial to the overall success of the practice and to ensuring that APPs continue to contribute and thrive in the oncology arena. *Methods:* We surveyed APPs and nurses to assess whether and how their contributions were being measured. An overview of the tools currently used in clinical practice is also provided. *Results:* The survey had an approximately 10% response rate and was answered by 59 APPs (who constituted 80% of the respondents). APP productivity was formally (36%) or informally (42%) measured for the majority of respondents, but only 25% believed that their productivity was being measured accurately. Relative value units (RVU, 39%) and hours per patient day (19%) were used; however, most APPs considered RVU an ineffective measure of both productivity and value. Value was not measured for many respondents (46%), and 29% did not know whether their value was being assessed. This is despite the fact that respondents spent an average of 19.8 hours per week on value-added activities that improve patient care and satisfaction. *Conclusions and Recommendations:* The crux of the issue is whether APPs can realistically gauge their value and contribution to a practice if the care they provide for their patients is not accurately assessed. APPS should ask whether they feel valued by their practice, patients, and physicians and how this impacts their overall job satisfaction. These answers could be important for retention of APPs and should be used to initiate conversations to increase awareness of APP contributions to the practice. We recommend that APPs consider tracking their work activity and the time it takes to complete tasks, especially those that are non-billable services. APPs should also consider asking the practice to review productivity of the team before and after the APP joined the practice to raise awareness of their contributions.

## JL420. Redesigning How We Communicate Important Safety Information (ISI) Using the Universal Patient Language

Elizabeth M. Chebli, PharmD, CMPP, Bristol-Myers Squibb, New York, NY

*Background:* The Universal Patient Language (UPL), is a set of resources developed by Bristol-Myers Squibb, to help communicate with patients about complex topics. At the core of the UPL are seven foundational principles. BMS applied these principles to create a UPL version of Important Safety Information (ISI). Before and during oncology treatment, patients need to understand the treatment’s risks, benefits, and other important information on how to take the medicine and drug interactions. Pharmaceutical companies communicate these details through the ISI. Traditionally, ISI is developed within a fixed, text-only template working within specific FDA guidance. The goal was to redesign how ISI is communicated by applying the UPL, to make it more inviting for patients to read, and potentially easier to understand. We started with a specific drug’s ISI, with the goal of using the output as a starting point for redesigning ISI for other BMS medicines. *Method:* Co-creation, the core method of applying the UPL, is a service-design technique that brings together multiple perspectives to reimagine and build new materials. To design a UPL version of ISI, a co-creation session was held with patients, communication experts and BMS employees with knowledge of the relevant regulations. Together, participants identified ways to improve ISI, and built rapid prototypes of what a UPL ISI could look like. The prototypes that emerged were further refined and validated with patients and BMS stakeholders. *Results/outcome:* The first UPL ISI was included in patient-facing brochures and released in 2015. The UPL redesign is much more visual, using whitespace, icons and simpler language. It also includes additional information, based on what patients said was important to them at the co-creation. To date, three different BMS products have implemented the UPL ISI. Enterprise templates and usage guides for ISI have also been created for other brands looking to use the UPL ISI. In small-scale testing, participants consistently responded positively to the design’s ability to help in identifying the most relevant information, and communicating the importance of following the instructions. These effects were stronger for respondents who had a treatment-appropriate diagnosis. In qualitative interviews, patients were overwhelmingly receptive to the new design—one patient said "I would put it on my fridge". *Implications:* The UPL tool can be used in oncology to help make complex information easier for patients to understand. The UPL ISI is just one application and other case studies and tools can be found on UPL.org.

## JL421. Review of a Genetic Risk Assessment Delivery Model in a Private Community Oncology Practice

Doreen Grzelak, NP-C, CBCN, MSN, AOCN®, Virginia Cancer Specialists, Washington, DC

According to the Oncology Nursing Society (ONS), part of the advanced practice nurse (APN) role is to provide genetic and genomic care to individuals in conjunction with expert providers (ONS, 2012). This is a review of a genetic risk assessment program in a private community oncology practice. The delivery model utilized includes physicians, an APN and a genetic counselor. Data was reviewed retrospectively from the inception of the program in 2011 through 2015. In addition to reviewing the number of patients seen, data was analyzed to determine the most frequent diagnosis, as well as results of genetic testing. We found that there was a need for this service for current patients in our practice and affected individuals in the community, as well as individuals in the community who were unaffected but had a family history of cancer. In 2014 and 2015 over one half of those seen were referred from outside of the practice. As expected, the most frequent diagnosis of those counseled was breast cancer. The second largest group was unaffected individuals seeking counseling based on family history. Additionally, as expected with the advent of panel testing, our results revealed an increase in variants of uncertain significance (VUSs). As the knowledge of genetic information continues to evolve, changes to guidelines for testing of individuals with cancer continues to expand. We anticipate the demand for genetic counseling to increase. The use of qualified non-genetic counselors in conjunction with genetic counselors in private community oncology practice is a delivery model that provides a needed service for oncology patients and their families. As a result, advanced practitioners (APs) will need to find methods to remain updated on recommendations for genetic risk assessment to provide counseling or refer patients appropriately.

## JL422. Screening for and Managing Distress in Patients With Metastatic Lung Cancer

Victoria Sherry, DNP, CRNP, ANP-BC, AOCNP®, Hospital of the University of Pennsylvania, Philadelphia, PA, Susan M. Schneider, PhD, RN, AOCN®, FAAN, Duke University School of Nursing, Durham, NC, Ranganathan Anjana, MD, Hospital of the University of Pennsylvania, Philadelphia, PA, and Carmen Guerra, MD, MSCE, FACP, Hospital of the University of Pennsylvania, Philadelphia, PA

Patients with metastatic lung cancer experience high levels of distress due to their disease trajectory and treatment. Oncology nurses are experts in patient care and symptom management giving them the opportunity to screen and treat patients’ distress. Screening patients for distress and managing their symptoms can have a substantial impact on quality of life, treatment adherence, clinical outcomes, and healthcare costs, and therefore, is recommended by the Commission on Cancer, National Comprehensive Cancer Network, Institute of Medicine, and Oncology Nursing Society. This quality improvement project was conducted to pilot distress screening using the National Comprehensive Cancer Network’s Distress Thermometer into thoracic oncology patient care and to evaluate the effect of a multifaceted intervention consisting of a patient education pamphlet and a nurse coaching call on distress levels. Screening identified 41/92 patients with distress levels > 4. This group had a mean distress score of 6.7436, (SD = 1.37109), with severe distress (score > 7) reported in 69.2% of patients. The number of symptoms reported ranged from 4 to 24. The most commonly reported symptoms that caused distress were fatigue, worry, pain and nervousness. A paired samples t-test revealed a significant decrease in distress scores following administration of a patient education pamphlet and a nurse coaching call. The results showed that management of distress through the use of this intervention can make a significant impact on reducing patient’s distress levels.

## JL423. Soft Tissue Sarcoma: A Case Study and Multidisciplinary Management

Allison L. Lock, MPAS, PA-C, MD Anderson Cancer Center, Houston, TX

*Introduction:* Soft tissue sarcoma (STS) is cancer of connective tissues and comprises a collection of over 50 distinct diagnoses. Approximately 11,000 cases are diagnosed in the US each year, accounting for about 1% of all adult cancers. Each histologic subtype of STS is considered rare and diagnosis can be challenging. STS can arise anywhere in the body, most frequently in the extremities. Initial presentation can mimic benign etiology, i.e. lipoma, as many patients complain of a painless lump. Clinicians ought to consider sarcoma in the differential diagnosis, as proper diagnosis is the cornerstone of effective treatment. Management of STS is complex and often requires a multidisciplinary approach. Depending on histology, NCCN Guidelines dictate that any combination of chemotherapy, radiation, and surgery may be warranted. *Purpose:* To highlight a rare case of STS and discuss multidisciplinary approach to sarcoma management. *Description:* A 72 year old male presents with a two-week history of a grape-sized painless mass in his left upper arm. The patient underwent piecemeal excision of the presumed lipoma. Pathology revealed a high grade sarcomatous lesion with positive margins. He was referred to a high-volume cancer center with a designated Sarcoma Center. Review of pathology revealed myxofibrosarcoma. He then underwent radiation followed by re-resection. Pathology revealed an infiltrating superficial spreading pattern without myxoid features, resulting in change of diagnosis to undifferentiated pleomorphic sarcoma (UPS) with superficial spreading features. The patient participated in a limb perfusion clinical trial. Approximately 6 weeks later, he noted a rapidly growing nodule near the surgical scar. He received one cycle of chemotherapy, which was poorly tolerated. The mass progressed rapidly into a multinodular coalescence measuring over 10 cm in aggregate. He is scheduled for shoulder disarticulation with curative intent. *Discussion:* Superficial spreading UPS is a rare variant of STS. Advanced Practice Providers (APPs) should consider STS when evaluating subcutaneous masses, as proper diagnosis is vital for appropriate management. In this case, initial attempts at resection were made prior to obtaining tissue diagnosis. The diagnosis was complicated by the piecemeal fashion of the initial resection. A multidisciplinary approach was used in the treatment of this patient and involved surgery, radiation, chemotherapy, and experimental therapy. Ultimately, the biology of the disease prevailed and resulted in the necessity of amputation as the only known means for potential cure. There are ample opportunities for APP involvement at every level of care from primary to tertiary care.

## JL424. The Interrelationship of Polypharmacy on Functional Status and Quality of Life in the Elderly Chronic Lymphocytic Leukemia Patient

Patricia Karwan, DNP, APRN-BC, Care New England/Women and Infant Hospital, Providence, RI

*Objective:* The purpose of this descriptive correlational study was to examine how the number of medications taken on a daily basis by elderly Chronic Lymphocytic Leukemia (CLL) patients is related to their functional status and quality of life. No previous studies have been identified that have explored the relationship between polypharmacy, quality of life, and functional status in CLL patients. Sample/*Method:* Sixty four elderly CLL patients in Rhode island completed a one time, descriptive, survey. This study examined five key areas related to these variables: (a) demographic characteristics of elderly CLL patients, (b) level of functional status in elderly patients with CLL, (c) level of quality of life of elderly patients with CLL, (d) the relationship of functional status and quality of life of elderly CLL patients and finally, (e) the relationship between quality of life and functional status grouped by the number of medications. Functional status was measured using the Lawton IADL scale. Quality of life was measured using the Quality Life Scale. Demographic information was collected with a 7-item survey. Statistical testing was done to analyze the demographic data and determine if any relationships were found among the variables. *Results:* There were significant and positive relationships in the two medication groups, demonstrated by the results. In the group where patients were taking 3-5 medications, the functional status scores were associated with higher quality of life scores. Functional status explained a 45% variance on the quality of life. The last group of patients taking more than 5 medications had a similar positive relationship where the higher functional status scores were associated with a better quality of life score. Conclusion: Overall, while the results were positive and significant, it appears that medications do affect the functional status and the quality of life in elderly CLL patients. There was a statistical significance and positive relationship with patients taking 3-5 medications, and patients taking greater than 5 medications. Functional status scores were associated with higher quality of life. Implication: This research study will contribute to the limited existing knowledge about the complex interactive effects of polypharmacy on the functional status and quality of life in the elderly CLL patients. Advance practice nurses in the oncology field are in a unique position to offer suggestions and to help manage symptoms, assess quality of life and functional status in the above patients. Early assessment of the patient functional baseline leads to better patient care.

## JL425. Tumor Lysis Syndrome: An Electrolyte Emergency

Jan Garza-Dennis, RN, MSN, ANP-C, AOCNP®, University of Texas at Houston, MD Anderson Cancer Center, Houston, TX

*Objective:* Tumor lysis syndrome (TLS) refers to the constellation of metabolic disturbances that may follow the initiation of cancer treatment. It can occur in patients with bulky, rapidly proliferating, treatment-responsive tumors such as high-grade lymphomas, acute leukemias, Burkitt’s Lymphomas, as well as in chronic leukemias with high white cell counts. TLS results from the release of intracellular ions and metabolic byproducts into the systemic circulation that contribute to electrolyte abnormalities resulting in acute systemic symptoms and potentially death if not identified and managed early. This poster will illustrate an evidence-based approach to the assessment, identification and management of TLS by advanced practice providers (APPs). *Methods:* An evidence-based algorithm is utilized for the identification, treatment and monitoring of tumor lysis syndrome at a National Cancer Institute-designated comprehensive cancer center. Application of this algorithm will be presented in the context of a case study, including diagnostic testing and treatment protocols. The case study focuses on a 48 year old man with T Cell prolymphocytic leukemia who presented with hyperkalemia, hyperuricemia, hyperphosphatemia, elevated BUN and creatinine and hypocalcemia. *Results:* Patient JG was identified as high risk according to the algorithm with hyperleukocytosis, LDH greater then 2 x normal, uric acid greater then 7.5 and high risk disease. JG was treated with allopurinol due to 6-PD Deficiency. By day 6 this patient’s lab work indicated TLS and AKI, suggesting need for aggressive continuous renal replacement therapy (CRRT). Clinically the patient was very advanced in TLS/AKI and due to G6PD Deficiency, we were not able to treat with rasburicase. Implementation of an evidence-based algorithm can be applied for the successful treatment and management of TLS, a potentially life-threatening oncologic emergency. The case study demonstrates how application of the algorithm resulted in care of the patient. *Conclusions:* Patients with TLS are very critical and must be assessed early and frequently to reduce mortality. Application of algorithms for the identification, treatment and monitoring of TLS is a fundamental component of successful APP practice in managing this potentially life-threatening condition. *Recommendations:* Hematology APPs should be familiar with early signs of TLS and manage patients, preferably utilizing evidence-based algorithms.

## JL426. The Impact of the ONc-PoWER Course on the Professional Development of Nurse Practitioners

Margaret Quinn Rosenzweig, PhD, FNP-BC, AOCNP®, FAAN, Sara Jo Klein, MS, BSN, RN, Mary Connolly, BSN, RN, and Rose Hoffmann, PhD, RN, University of Pittsburgh School of Nursing, Pittsburgh, PA

*Background:* The Oncology Nurse Practitioner Web Education Resource (ONc-PoWER) is an online course developed by Margaret Rosenzweig, PhD, FNP-BC, AOCNP® from the University of Pittsburgh School of Nursing and funded by the National Cancer Institute (1 R25 CA148050-01A1). The course is designed specifically for Nurse Practitioners (NPs) in their first year of oncology practice paired with an on-site mentor (physician, nurse practitioner or physician assistant). The 5th module in the course focuses on Self-Care and Professional Development. The purpose of this study was to examine the impact of the ONc-PoWER course on the professional development of Nurse Practitioners 6 months or more after course completion. *Method:* NPs and mentors completed the course over 4-6 months. As a follow up measure 51 NPs were sent a survey regarding their professional activities since the completion of the ONc-PoWER course. There were 9 items with Likert scale responses of: 3) To a great degree, 2) Somewhat, 1) Not at all. *Results:* There were n=24 (41%) response. All but one were female with 23 white/Caucasian and 1 black/African American. See table. Conclusion: The ONc-PoWER course was effective in encouraging Oncology Nurse Practitioners in their professional development. Most effective was utilization of evidence for practice; least effective was the translation of evidence for other health care providers. This is an ongoing analysis. *Implications:* Future renditions of the course will include a stronger emphasis on scholarly publications and the translation of research findings and evidence.

**Table:**
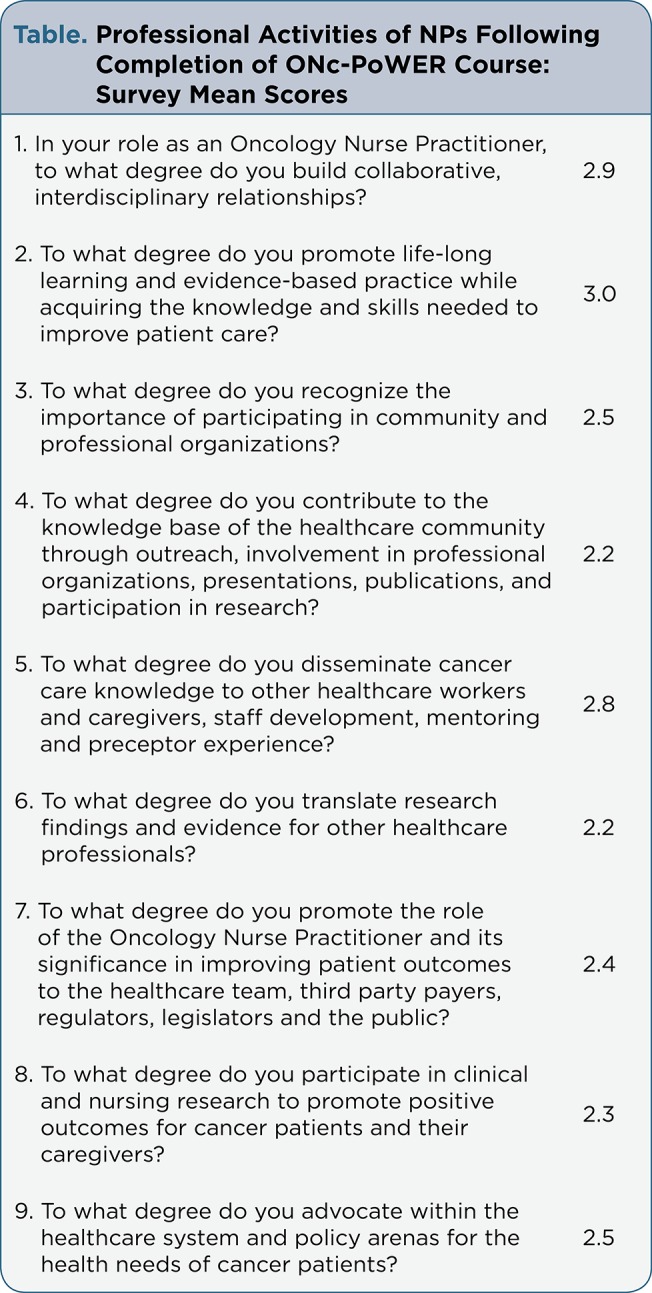
Professional Activities of NPs Following Completion of ONc-PoWER Course: Survey Mean Scores

## JL427. The Effect of Prophylactic Naproxen, Prophylactic Loratadine, or No Prophylaxis on Bone Pain in Patients With Breast Cancer Receiving Chemotherapy and Pegfilgrastim

Andrew Guinigundo, MSN, RN, CNP, ANP-BC, Oncology Hematology Care Inc., Cincinnati, OH, Linda Vanni, MSN, RN-BC, ACNS-BC, NP, Providence Hospital, Washington, D.C., Cathy Maxwell, BSN, RN, OCN®, TESARO Inc., Waltham, MA, Maureen Reiner, MA, MS, Amgen Inc., Thousand Oaks, CA, Jacob Garcia, MD, Amgen Inc., Thousand Oaks, CA, Phuong Khanh Morrow, MD, Amgen Inc., Thousand Oaks, CA, and Jeffrey Kirshner, MD, Hematology-Oncology Associates of Central New York, East Syracuse, NY

*Background:* Mild-to-moderate bone pain is the most commonly reported adverse event (AE) associated with pegfilgrastim. Anecdotal reports and some studies have suggested that naproxen (an NSAID) and loratadine (an antihistamine) may help reduce bone pain, and both agents are widely used in the clinic for this purpose. Data from prospective clinical trials on the efficacy of these interventions are limited. *Methods:* In this open-label study (NCT01712009), women ≥18 years of age with newly diagnosed stage I-III breast cancer and ECOG performance status ≤2 who were planning ≥4 cycles of adjuvant or neoadjuvant chemotherapy with pegfilgrastim support starting in cycle 1 were randomized 1:1:1 to receive prophylactic naproxen, prophylactic loratadine, or no prophylaxis to prevent bone pain. Patients who were randomized to the active treatment arms received naproxen (500 mg twice daily) or loratadine (10 mg once daily) for 5 days beginning on the day of pegfilgrastim administration in each of the first 4 chemotherapy cycles. Pegfilgrastim (6 mg) was administered once per cycle, 24-72 hours after chemotherapy. The primary endpoint of the study was all-grade bone pain in cycle 1 from AE reporting. Secondary endpoints included bone pain in cycles 2-4 and across all cycles from AE reporting and various measures of patient-reported bone pain by cycle and across all cycles. *Results:* Six hundred patients were enrolled. Race: white 83.0%, black or African American 14.1%, Asian 1.2%. Mean (SD) age: 54.2 (11.1) years. Stage I/II/III: 25.6%/50.1%/24.4%. The percentage of patients with all-grade bone pain in cycle 1 from AE reporting was lower in the naproxen and loratadine arms than in the no prophylaxis arm, but these differences were not statistically significant. Mean, maximum, and AUC for patient-reported bone pain were lower in the naproxen and loratadine arms than in the no prophylaxis arm, and some of these differences were statistically significant (no multiplicity correction). Loratadine was associated with fewer AEs than naproxen, and fewer patients discontinued loratadine. See table for additional results. *Conclusions:* Levels of all-grade bone pain from AE reporting were comparable in the naproxen, loratadine, and no prophylaxis groups, but there was a consistent trend toward lower pain with naproxen and loratadine, particularly when analyzing measures of patient-reported pain. Loratadine was better tolerated than naproxen. *Recommendations:* Given its ease of administration, tolerability, and potential benefit, treatment with daily loratadine to help prevent bone pain should be considered for patients receiving chemotherapy and pegfilgrastim.

**Table:**
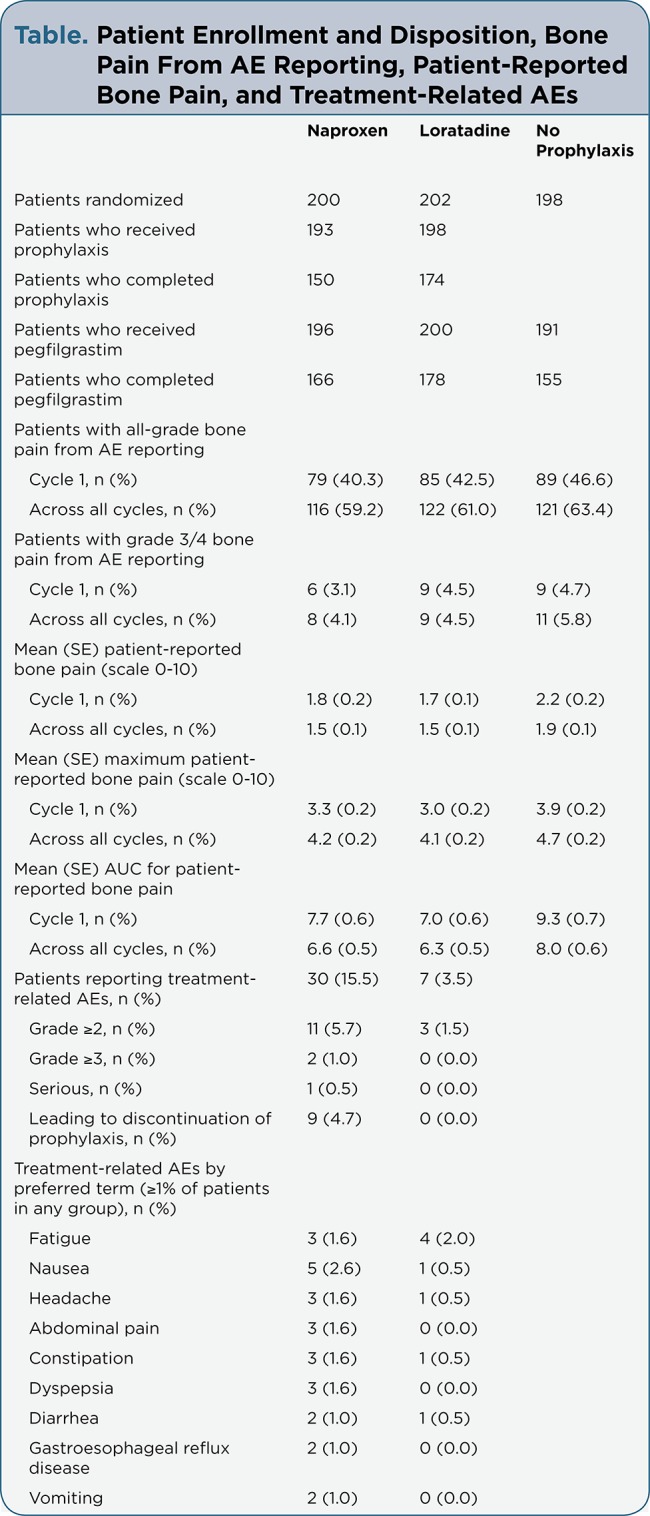
Patient Enrollment and Disposition, Bone Pain From AE Reporting, Patient-Reported Bone Pain, and Treatment-Related AEs

## JL428. Symptoms and Management of Cerebral Progression in Patients With Anaplastic Lymphoma Kinase-Positive (ALK+) Non-Small Cell Lung Cancer (NSCLC): A Clinical and Nursing Perspective

Rachael Pollina, MS, MSN, FNP-BC, Memorial Sloan Kettering Cancer Center, New York, NY, Wendi Stone, RN, NP-C, University of Texas MD Anderson Cancer Center, Houston, TX, Kate Kegg, MPAS, PA-C, University of Texas MD Anderson Cancer Center, Houston, TX, and Anne S. Tsao, MD, University of Texas MD Anderson Cancer Center, Houston, TX

*Background:* Around 30–50% of ALK+ NSCLC patients have brain metastases (BM) before initiating ALK inhibitor (ALKi) treatment. In general, the prognosis for patients with BM is poor (median survival 7 months). Standard treatments include surgery, stereotactic radiosurgery (SRS), and whole-brain radiotherapy (WBRT). However, patients with ALK+ NSCLC and BM are a unique group, with prolonged survival when treated with SRS/WBRT and ALKis. Up to 50% of ALK+ NSCLC patients will have BM at presentation or at time of first progression. With second/thirdgeneration ALKis that cross the blood–brain barrier, intracranial disease control can be temporarily achieved. However, cerebral progression can eventually occur (0–21% across second-generation ALKi clinical studies). It is well established that the number of metastatic CNS sites significantly impacts prognosis and treatment options. Delayed detection of progression may result in increased disease burden and decreased survival. Therefore, routine surveillance with vigilant CNS monitoring is required to facilitate early identification of patients with disease progressing in the brain. The signs and symptoms of cerebral progression include:

- Headaches
- Nausea/vomiting
- Weakness
- Progressive neurologic deficit (e.g. partial paralysis, speech impediments)
- Seizures
- Sensory defects (e.g. visual impairment)
- Gait abnormalities
- Cognitive impairment (confusion/memory loss)

Treatment strategies include dexamethasone for edematous and symptomatic BM, and anticonvulsants for seizure prevention. It is important to distinguish the effects of new BM from radiotherapy-induced radionecrosis, as this can present as an edematous growing mass on imaging. Likewise, some ALKi side effects can be difficult to differentiate from symptoms of new BM (e.g. visual disturbances, nausea/vomiting). The side effects of treatments for symptoms must also be managed; strategies include minimizing/tapering corticosteroid doses and adjustment of anticonvulsants based on altering symptoms. *Recommendations:* As soon as symptoms of BM are recognized, primary oncology healthcare providers should reach out to neurosurgeons/neurologists/neuro-oncologists and CNS radiotherapists as appropriate. ALK+ NSCLC patients require regular interval followups for focused neurologic assessment with recent imaging results. Ophthalmologists should be consulted to manage visual disturbance. Physical therapy may help manage aerobic incapacity, weakness, balance/coordination, and visual-perceptual impairment. Initiation of BM treatment, including switching ALKi therapy, should be considered (it is recommended that ALKis are held during WBRT). *Conclusions:* Early identification of CNS progression has the potential to improve ALK+ NSCLC patient prognosis by providing an opportunity for rapid and timely intervention. Repeated treatment of CNS disease with ALKis/SRS is becoming the new standard of care, with ALKis preferred over WBRT. 

## JL429. Transfusion Practice Patterns in Patients Receiving Myelosuppressive Chemotherapy for Nonmyeloid Cancer: Results From a Prospective Observational Study

Chet Bohac, MD, PharmD, Amgen Inc., Thousand Oaks, CA, James Granfortuna, MD, Cone Health, Greensboro, NC, Kaye Shoffner, BSN, MHA, OCN®, Alamance Regional Cancer Center Research, Cone Health, Burlington, NC, Stephen E. DePasquale, MD, University of Tennessee College of Medicine, Chattanooga, TN, Xingwei Sui, MD, PhD, Providence Regional Cancer System, Lacey, WA, Sejal Badre, PhD, Amgen Inc., Thousand Oaks, CA, and Cisio De Oliveira Brandao, MD, MBA, MsC, Amgen Inc., Thousand Oaks, CA

*Background:* The decision to prescribe a packed red blood cell (PRBC) transfusion in patients with chemotherapy-induced anemia includes assessment of clinical features such as the patient’s cancer type and treatment regimen, severity of signs and symptoms of anemia, and presence and severity of comorbidities. Here, we sought to examine contemporary transfusion practices in patients receiving myelosuppressive chemotherapy for nonmyeloid cancer. *Methods:* Eligible patients were ≥18 years of age, had nonmyeloid cancer, were receiving first- or second-line myelosuppressive chemotherapy, had baseline hemoglobin (Hb) ≤10.0 g/dL, and were planned to receive ≥1 PRBC transfusion. Key exclusion criteria were receipt of an erythropoiesis-stimulating agent within 8 weeks prior to screening and/or chronic renal insufficiency. The study ran from 30-Sep-2014 to 31-Oct-2015. Data were collected from patients’ medical records, laboratory values, and physician/provider questionnaires to determine considerations for transfusions. The proportions of primary clinical considerations leading to PRBC transfusions and 95% exact binomial confidence intervals (CIs) were estimated. *Results:* The study enrolled 154 patients at 18 sites in the US. Of these, 147 (95.5%) received a PRBC transfusion (Table 1). Mean (95% CI) last Hb value before PRBC transfusion was 8.1 (8.0–8.3) g/dL. Fatigue was the most common patient-reported symptom affecting the decision to prescribe a PRBC transfusion (94 [64.4%] patients), followed by dyspnea on exertion (24 [16.4%] patients) (Table 2). The most frequently reported primary clinical consideration for prescribing a PRBC transfusion was anemia symptoms (106 [72.1%] patients; mean Hb, 8.1 g/dL), followed by Hb value (37 [25.2%] patients; mean Hb, 8.1 g/dL) and medical history (4 [2.7%] patients; mean Hb, 8.5 g/dL). Overall, 70 (47.9%) patients received a transfusion at Hb ≥8.0 g/dL. Consistent results were observed when data were analyzed by age ( <65 vs ≥65 years), sex, tumor type, comorbid conditions, chemotherapy type/line, and site type (academic vs nonacademic). Comorbid medical conditions were reported in 41 (27.9%) patients. Only 9 (6.1%) patients had underlying cardiovascular disease. Conclusion: In this study, the primary clinical considerations for prescribing a PRBC transfusion were anemia symptoms in 72.1% of patients, Hb value in 25.2% of patients, and medical history in 2.7% of patients; 47.9% (70 patients) received a transfusion at Hb ≥8.0 g/dL. Results show that multiple clinical factors, not just the Hb value, were used in the decision to prescribe PRBC transfusions in this patient sample. 

**Table 1:**
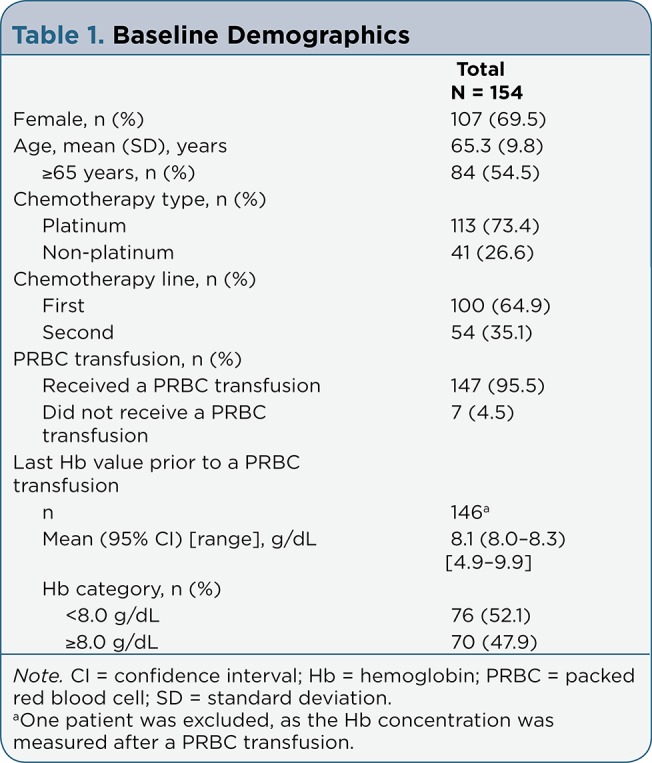
Baseline Demographics

**Table 2:**
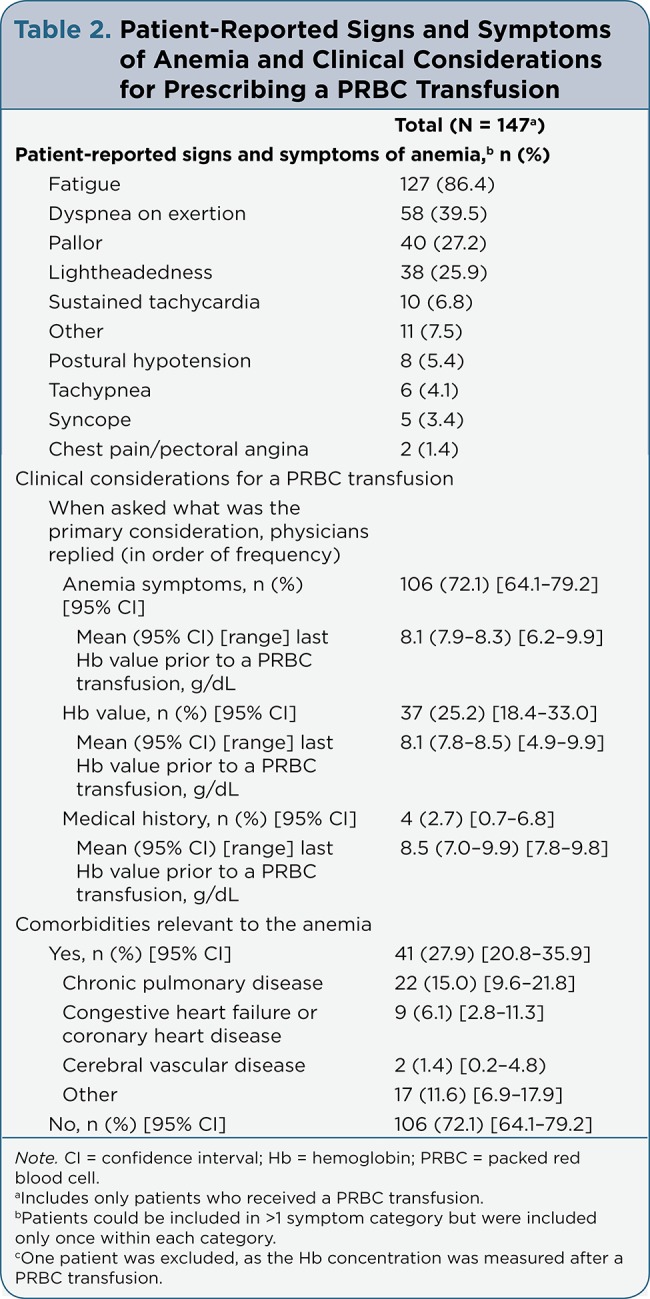
Patient-Reported Signs and Symptoms of Anemia and Clinical Considerations for Prescribing a PRBC Transfusion

